# Prevalence and Associated Factors of Depressive Symptoms in Patients with Myasthenia Gravis: A Cross-Sectional Study of Two Tertiary Hospitals in Riyadh, Saudi Arabia

**DOI:** 10.1155/2019/9367453

**Published:** 2019-09-15

**Authors:** Mohammed H. Alanazy

**Affiliations:** Department of Internal Medicine, King Saud University Medical City and College of Medicine, King Saud University, Riyadh, Saudi Arabia

## Abstract

**Objectives:**

This study is aimed at elucidating the prevalence of depression in patients with myasthenia gravis (MG) and examining the risk factors associated with depression.

**Methods:**

We evaluated adult patients with MG who were recruited from two tertiary hospitals in the central region (Riyadh) of Saudi Arabia. Data were collected with a two-part standardized questionnaire: the first part included data on sociodemographic and clinical features of MG including disease type and duration, therapies, prednisolone dose, time of the last relapse, previous critical care unit admissions, MG status (controlled, partially controlled, or uncontrolled), and comorbid diseases; the second part included items from the previously validated Arabic version of the Patient Health Questionnaire-9 (PHQ-9).

**Results:**

In total, 104/150 (69.3%) patients participated (72 females) with a mean age of 38.0 ± 16.0 years. The mean PHQ-9 score was 7.02 ± 6.1. Among all the participants, 27 (26.0%) patients had depression (PHQ‐9 ≥ 10). Multiple logistic regression analysis revealed that uncontrolled MG status (OR = 12.31, 95%CI = 1.13‐133.8, *P* = 0.04) was the only factor independently associated with depression. Collectively, the prevalence of depression among patients of the primary care clinics (PCC) as reported by 5 previous studies across multiple regions of the country was 15.8%. The odds of depression among MG patients were twofold higher than those among PCC patients (OR = 2.05, 95%CI = 1.30‐3.22, *P* = 0.002).

**Conclusions:**

Approximately a quarter of MG patients have depression. Achieving a minimal manifestation or better MG status may decrease the depression rate in these patients.

## 1. Introduction

Myasthenia gravis (MG) is a chronic autoimmune disease involving neuromuscular junctions (NMJ) [1]. The cardinal feature of MG is fatigable weakness of the affected muscles with the following symptoms: ptosis, diplopia, dysphagia, dysarthria, dyspnea, and neck and limb muscle weakness [1]. Patients with MG are required to cope with the chronicity of the disease and often need lifelong therapy with single or combined MG-specific therapies [1]. Corticosteroids are currently the mainstay oral immunosuppressive therapy, which can be, when required, combined with steroid-sparing therapies [2]. Patients with refractory MG require maintenance therapy with intravenous immunoglobulin, plasmapheresis, or eculizumab [2, 3]. In addition to the side effects of these therapies, some of them require regular admissions (every 2–4 weeks) to a daycare unit to be administered [2, 3]. Therefore, MG has a significant impact on patients' daily activities and health-related quality of life (HRQoL) [4].

Several studies have investigated the prevalence of depression in patients with MG and reported inconsistent results [5–13]. A study using a structured psychiatric interview (MINI-plus) estimated the prevalence of depression to be 26.1% [5], while another study that used a semistructured interview estimated the rate of affective disorder to be 32.0% [6]. Using self-reported scales of depression, the prevalence of depression has been estimated to be 13.6% in Japanese patients with MG using the Beck Depression Inventory second edition (BDI-II) scale [7] and 27.5% in Brazilian patients using the Hospital Anxiety Depression (HAD) scale [8]. Using the Hamilton Depression Rating Scale, Aysal et al. reported that 50.0% of Turkish patients with MG have depression [9]. Another study reported higher scores on the BDI in patients with MG compared to healthy controls; however, their scores did not exceed the cut-off score for depression [10]. Fisher et al. reported a 33% rate of depression among patients with MG, which was higher than the rate in the general population in the USA but similar to the rate in patients with other chronic diseases [11]. In contrast, Hoffmann et al. reported that the rate of depression among patients with MG (19.6%) was comparable to the rate in the German general population [12]. This was also supported by Paul et al. who observed no difference in the rates of depression of patients with MG and a control group on the evaluative and mood subscales of the Chicago Multiscale Depression Inventory (CMDI) [13].

The prevalence of mood disorders varies across countries and cultures; however, it has not yet been studied in patients with MG in Saudi Arabia. Therefore, this study sought to elucidate the prevalence of depression among patients with MG in Saudi Arabia and to determine the risk factors associated with depression.

## 2. Methods

### 2.1. Participants and Setting

The study was conducted at two tertiary hospitals (King Saud University Medical City and Security Forces Hospital) in Riyadh, Saudi Arabia. Ethical approval was obtained from the respective institutional review boards at each center. Data obtained for this study were part of a larger cross-sectional study. Data were collected using an anonymous questionnaire, which was sent electronically to adult (age ≥ 18 years) patients with established MG immediately after their follow-up clinic visit. Patients who had missed their follow-up appointments were called by phone to obtain their permission to send them the electronic link of the survey. We only included patients with an established diagnosis of MG by a neurologist based on the presence of clinical features of MG and at least one of the following: antiacetylcholine receptor antibodies, antimuscle-specific tyrosine kinase antibodies, decremental response on repetitive nerve stimulation of at least 10%, increased jitter or blocking on single-fiber electromyography in at least 3 fiber pairs, and unequivocal improvement with pyridostigmine or immunosuppressive therapy. Patients with intellectual disabilities were excluded. The first page of the questionnaire explained the study purpose and asked for consent to participate. Only patients who clicked the “agree to participate” button were permitted to continue the survey. The study commenced on March 1, 2018, and ended on March 15, 2019.

### 2.2. Study Measures

The electronic survey collected data on sociodemographic and clinical features of MG including disease duration, type of MG (ocular or generalized), MG therapies, prednisolone dose, time of the last MG relapse, previous critical care unit (ICU) admission, patient-reported MG status (controlled, partially controlled, or uncontrolled), and comorbid diseases.

Depression was assessed with the validated Arabic version of the Patient Health Questionnaire-9 (PHQ-9) [14]. The PHQ-9 has been developed and validated as a self-report screening tool for depression in the primary care setting [15]. The PHQ-9 items are scored on a Likert scale with item score ranging from 0 to 3. A total score ≤ 4 indicates “minimal or no depression,” 5-9 “mild depression,” 10-14 “moderate depression,” 15-19 “moderately severe depression,” and 20-27 “severe depression” [15]. The PHQ-9 has been used as a screening tool for depression in patients with different neurological diseases [16–18].

### 2.3. Historical Control Group

We searched MEDLINE (January 2000 to April 2019) using two pairs of combined search terms “PHQ” and “Saudi Arabia” or “depression” and “Saudi Arabia.” We also screened the references of the selected articles to identify additional eligible articles. Only studies that had reported the percentages of Saudi adult patients with moderate or severe depression (PHQ‐9 ≥ 10) were included. The identified studies were categorized into those for patients attending primary care clinics (PCC) and those for patients with other disorders.

## 3. Analysis

Descriptive statistics, frequencies, and proportions were used to analyze demographic and clinical data, as appropriate. Patients were categorized into two groups based on the total PHQ-9 score: “depression” (PHQ‐9 ≥ 10) and “no depression” (PHQ‐9 < 10) [15]. Chi-square test, Kruskal-Wallis test, Mann-Whitney *U* tests, and Student's *t*-test were used to compare variables between the “depression” and “no depression” groups, as appropriate. Variables that showed significant association with depression were entered in a multiple logistic regression model; we controlled for age and sex. Patient-reported MG statuses “controlled” and “partially controlled” were grouped and compared to the “uncontrolled” status. Odds ratios (OR) and 95% confidence intervals (CI) were used to assess the association between independent variables and “depression.” After extracting the numbers and percentages of patients with and without depression from the previous studies that had used the PHQ-9, OR and 95% CI were computed to compare the rate of depression in patients with MG with the rate of depression in patients with other disorders. The threshold for a significant two-tailed *P* value was set at <0.05. Data were analyzed with SPSS version 23 (IBM, Armonk, NY).

### 3.1. Patient and Public Involvement

Patients were involved by completing the study questionnaire. Patients were not further involved in the design or implementation of this study.

## 4. Results

Patients' demographics and MG characteristics are shown in Table 1. In total, 104/150 (69.3%) patients participated (72 females and 32 males) with a mean age of 38.0 ± 16.0 years. The mean PHQ-9 score was 7.02 ± 6.1. Among all participants, 27 (26.0%) patients had depression (PHQ‐9 ≥ 10). Univariate analysis revealed that higher level of education, higher family income, and patient-reported uncontrolled MG status were associated with depression, whereas longer MG duration (≥4 years) was less associated with depression (Table 1). However, only “uncontrolled” MG status was independently associated with depression in the multiple logistic regression model (OR = 12.31, 95%CI = 1.13‐133.8, *P* = 0.04) (Table 2).

Table 3 shows the odds of depression in patients with MG in this study compared to those in PCC patients and patients with other medical disorders reported in selected studies. We identified 5 cross-sectional studies that used the PHQ-9 to determine the prevalence of depression in PCC patients, two of which were conducted in Riyadh (central province), two in Alkhobar (the eastern province), and one in Sharurah (the southern province). These studies involved a total of 2,228 adult patients, and the prevalence of depression was 15.8% based on a PHQ-9 score of ≥10. The prevalence of depression was significantly higher in our MG cohort than in the PCC patients included in the 5 studies (OR = 2.05, 95%CI = 1.30‐3.22, *P* = 0.002) (Table 3).

## 5. Discussion

The rate of depression among patients with MG in this study is generally comparable to those reported by studies in other countries [5–13]. While some previous studies have reported a higher rate of depression in patients with MG compared to the general population [11], other studies reported no difference [12, 13]. Since we did not have a healthy control group, we compared our results to those reported by previous studies on PCC patients in Saudi Arabia. To minimize bias arising from the use of different self-reported depression scales, we decided to include only studies that had used the PHQ-9 as a measure of depression. The odds of depression in patients with MG in this study were twofold higher than the corresponding average from all the included previous studies on PCC patients. Among these studies, the rate of depression reported by Abdelwahid et al. was the lowest (Table 3) [22]. Although the authors did not provide a clear justification for their findings [22], we speculate that the low rate might be related to the socioeconomic status of the study population who were recruited from a PCC in a secondary hospital and the geographical location and small size of the city in which the study was conducted. Another possible reason could be that the mean age of the participants was relatively lower than that in the other selected studies [14, 20, 21, 23].

We cannot exclude heterogeneity of the population recruited by the selected previous studies [14, 20–23], and therefore, it might have influenced our results. Not all the selected studies reported exclusion criteria [14, 20–23]. Patients with cognitive dysfunction may not be able to complete self-administered questionnaires accurately and are often assumed to have been excluded. However, only two studies explicitly excluded them [14, 22]. The other studies excluded patients with established diagnoses of psychiatric illnesses [14], depression [20], and unstable medical condition [22]. As expected, PCC patients include patients with chronic diseases such as diabetes, hypertension, and dyslipidemia. The rate of chronic diseases in this study (43.3%) was higher than that in the PCC patients reported by Abdelwahid et al. (10.7%) [22] and Aldabal et al. (15.7%) [21]. On the contrary, all patients included in the study by Alkhathami et al. had chronic diseases (diabetes and/or hypertension) and their mean age was higher than that in our study (50.9 ± 11.7 years); however, the odds of depression were significantly less than those in our study [23]. Therefore, it seems that the presence of chronic diseases does not explain the higher odds of depression observed in this study compared to those with lower percentages of chronic diseases. In addition, the rate of depression in MG patients in this study is comparable to the rate of depression among Saudi patients with sickle cell disease and gastrointestinal disorders [24, 25], but significantly less than that among patients with hematological malignancies and multiple sclerosis [26, 27].

This study examined the associations between sociodemographic and clinical parameters of MG and self-reported depressive symptoms. Univariate analysis revealed four variables that were associated with depression. Patient-reported uncontrolled MG status, higher level of education, and higher family monthly income were associated with depression, whereas longer MG duration was less associated with depression. Multiple logistic regression analysis revealed that only patient-reported uncontrolled MG status was independently associated with depression. This finding is similar to that of other studies that reported significant associations between MG severity and depression [7, 9]. However, other variables, such as dose of prednisolone and MG duration (early disease stage), which were reported by Suzuki et al. to be associated with depression [7], did not show independent associations with depression in this study. In addition to differences in the self-report scales used to measure depressive symptoms, other possible reasons for this discrepancy might be the smaller sample size and higher average prednisolone dose in this study compared to those of Suzuki et al. [7]. It is also possible that racial and cultural factors influenced our results to differ from those reported in the Japanese population. Other variables that have been previously shown to be associated with depression, such as unchanged MG status despite therapy [7] and stressful life events [9], were not evaluated in this study. However, we acknowledge that the former variable is probably embedded in the construct of the patient-reported uncontrolled MG variable used in this study.

The findings of this study, collectively with those of the previous studies, suggest that achieving MG control (minimal manifestation or better status according to the Myasthenia Gravis Foundation of America classification) with the lowest dose possible of corticosteroids will not only improve HRQoL but also decrease depressive symptoms [7, 28]. This places more emphasis on the need to use steroid-sparing therapies, including biologic agents, to allow tapering of corticosteroids to the lowest dose possible in refractory MG patients.

The association between depression and MG can be explained by many factors including the negative impact of MG on HRQoL [28]. MG is a chronic disease that limits patients' physical and social activities and the ability to enjoy hobbies [4]. In addition, ptosis, ocular misalignment, and nasal speech might also cause social withdrawal [29, 30]. Patients with uncontrolled MG are usually on a high-dose prednisolone; the side effects of which, especially weight gain and changes in facial expression, may increase the risk of depression [7]. Even mild MG can negatively impact patients' social life and the ability to enjoy pleasurable activities, which forces them to constantly make plans to overcome these limitations [4]. The relationship between MG and depression appears to be bidirectional. Previous studies have shown that depressive state is associated with a higher prevalence of fatigue (subjective feeling of exhaustion), which negatively impacts activities of daily living of patients with MG [12, 31].

This study has a few limitations. The cross-sectional design of this study hindered the establishment of a predictive model for the PHQ-9 among patients with MG; however, multiple logistic regression analysis demonstrated that patient-reported uncontrolled MG status was independently associated with the self-reported depressive symptoms. Another limitation is that we could not establish a temporal relationship between depression and MG status as we could not determine the number of patients that had depression preceding or following MG diagnosis. Furthermore, the use of self-reported questionnaires as a measure of depression may not accurately reflect the prevalence of depression among patients with a specific disease in a tertiary clinical setting. The use of electronic survey limited our ability to capture responses from illiterate patients and from those who do not use smartphones.

In conclusion, the rate of depression in patients with MG in Saudi Arabia is higher than that in primary care clinic patients and comparable to that reported in patients with MG by studies in other countries. Future studies using a structured psychiatric interview as a diagnostic test for depression are needed to validate these findings.

## Figures and Tables

**Table 1:** Demographic and clinical features sorted by PHQ-9 group.

Variables	Comments	All patients *N* = 104	PHQ-9		
			Score < 10(none & mild depression) *N* = 77	Score ≥ 10(moderate & severe depression) *N* = 27	*P* value
Age	M ± SD, year	38.0 ± 16.0	39.0 ± 16.8	35.1 ± 13.2	0.28
Sex	Male	32 (30.8)	25 (32.5)	7 (25.9)	0.53
	Female	72 (69.2)	52 (67.5)	20 (74.1)	
Married	Yes	68 (65.4)	47 (61.0)	21 (77.8)	0.12
Education	≤high school	53 (51.0)	44 (57.1)	9 (33.3)	**0.03**
	>high school	51 (49.0)	33 (42.9)	18 (66.7)	
Employment	Yes	34 (32.7)	23 (29.9)	11 (40.7)	0.3
Family monthly income	<10,000 SR^∗^	69 (66.3)	56 (72.7)	13 (48.1)	**0.02**
	≥10,000 SR^∗^	35 (33.7)	21 (27.3)	14 (51.9)	
MG type	Ocular	22 (21.2)	18 (23.4)	4 (14.8)	0.35
	Generalized	82 (78.8)	59 (76.6)	23 (85.2)	
MG duration	≥4 years	56 (53.8)	46 (59.7)	10 (37.0)	**0.04**
MG therapies	≥2	78 (75.0)	56 (72.7)	22 (81.5)	0.37
Prednisolone	Yes	74 (71.2)	52 (67.5)	22 (81.5)	0.17
Average 3-month prednisolone dose	M ± SD mg	12.6 ± 14.9	11.4 ± 14.8	16.2 ± 14.7	0.07
MG status^†^	Uncontrolled	5 (4.8)	1 (1.3)	4 (14.8)	**0.009** ^**‡**^
	Partially controlled	77 (74.0)	57 (74.0)	20 (74.1)	
	Controlled	22 (21.2)	19 (24.7)	3 (11.1)	
Last MG relapse	<1 year ago	42 (40.4)	27 (35.1)	15 (55.6)	0.06
Comorbidities	≥1	45 (43.3)	34 (75.6)	11 (24.4)	0.38
ICU admission	Yes	42 (40.4)	29 (37.7)	13 (48.1)	0.34

^∗^SR: Saudi riyal. The number 10,000 SR (≈2,666 $) represents the estimated median monthly income for the general Saudi population according to the 2013 Saudi household income survey [19]. ^‡^*P* values reported are unadjusted. To account for multiple tests using Bonferroni adjustment, here the statistically significant *P* value for a test is considered to be *P* < 0.017. ^†^*P* = 0.01 for comparisons of the rate of depression between the “uncontrolled” and “partially controlled” groups; *P* = 0.003 for comparisons between the “uncontrolled” and “controlled”; and *P* = 0.23 for comparisons between “the partially controlled” and “controlled” groups. Note: percentages reported in all rows reflect column percentages.

**Table 2:** Multiple logistic regression showing variables associated with depression among MG patients.

Variable	Comment	OR (95% CI)	*P* value
Age	Year	1.0 (0.97-1.03)	0.88
Sex	Male	0.63 (0.20-2.02)	0.44
Education level	>high school	2.03 (0.73-5.68)	0.18
Income	≥10,000 SR^∗^	2.25 (0.84-6.04)	0.11
MG duration	≥4 years	0.37 (0.13-1.10)	0.07
Patient-reported MG status	Uncontrolled	12.31 (1.13-133.8)	**0.04**

^∗^SR: Saudi riyal. The number 10,000 SR (≈2,666 $) represents the estimated median monthly income for the general Saudi population according to the 2013 Saudi household income survey [19].

**Table 3:** Odds ratio of depression among adult patients with MG compared to those in previous studies (note: rate of depression in MG = 26.0%).

	Clinic, city	Patients, *n*	Depression	OR (95% CI)	*P*
Studies in PCC					
Becker et al. [14]	PCC, Riyadh	431	20.0%	1.41 (0.86-2.31)	0.18
Al-Qadhi et al. [20]	PCC, Riyadh	477	18.9%	1.51 (0.92-2.47)	0.10
Aldabal et al. [21]	PCC, Alkhobar	680	16.0%	1.84 (1.13-2.98)	0.01
Abdelwahid and Al-Shahrani [22]	PCC, Sharurah	272	3.0%	11.57 (5.05-26.51)	<0.001
Alkhathami et al. [23]	PCC, Alkhobar	368	8.9%	3.56 (2.02-6.27)	<0.001
All above studies	PCC	2,228	15.8%	2.05 (1.30-3.22)	0.002
Other studies					
Alosaimi et al. [24]	GI, Riyadh	440	35.9%	0.63 (0.39-1.01)	0.054
Al Zahrani et al. [25]	SCD, Tabuk	89	36.0%	0.63 (0.34-1.16)	0.13
Abuelgasim et al. [26]	HM, Riyadh	211	46.4%	0.40 (0.24-0.68)	<0.001
Alosaimi et al. [27]	MS, Riyadh	163	46.6%	0.40 (0.24-0.69)	<0.001

CI: confidence interval; GI: gastrointestinal diseases; HM: hematological malignancies; OR: odds ratio; PCC: primary care clinics; MS: multiple sclerosis; SCD: sickle cell disease.

## Data Availability

Dataset is available upon written email request to the author.
